# An Experimental and Numerical Study of the Influence of Temperature on Mode II Fracture of a T800/Epoxy Unidirectional Laminate

**DOI:** 10.3390/ma15228108

**Published:** 2022-11-16

**Authors:** Yu Gong, Linfei Jiang, Linkang Li, Jian Zhao

**Affiliations:** 1 Chongqing Key Laboratory of Heterogeneous Material Mechanics, College of Aerospace Engineering, Chongqing University, Chongqing 400044, China; 2 State Key Laboratory of Coal Mine Disaster Dynamics and Control, Chongqing University, Chongqing 400044, China; 3 State Key Laboratory of Automotive Simulation and Control, Jilin University, Changchun 130022, China; 4 School of Civil Engineering, Chongqing University, Chongqing 400044, China; 5 School of Aerospace Engineering and Applied Mechanics, Tongji University, Shanghai 200092, China

**Keywords:** composite laminate, mode-II delamination, *R*-curve, temperature, cohesive law

## Abstract

Studies on mode II fracture have promoted the establishment of the delamination theory for unidirectional composite laminates at room temperature. However, under thermal conditions, the fracture behavior of composite laminates will exhibit certain differences. The delamination theory should be extended to consider the temperature effect. To achieve this goal, in this study, the mode II static delamination growth behavior of an aerospace-grade T800/epoxy composite is investigated at 23 °C, 80 °C and 130 °C. The mode II fracture resistance curve (*R*-curve) is experimentally determined. A fractographic study on the fracture surface is performed using a scanning electron microscope (SEM), in order to reveal the failure mechanism. In addition, a numerical framework based on the cohesive zone model with a bilinear constitutive law is established for simulating the mode II delamination growth behavior at the thermal condition. The effects of the interfacial parameters on the simulations are investigated and a suitable value set for the interfacial parameters is determined. Good agreements between the experimental and numerical load–displacement responses illustrate the applicability of the numerical model. The research results provide helpful guidance for the design of composite laminates and an effective numerical method for the simulation of mode II delamination growth behavior.

## 1. Introduction

Composite materials with high specific strength and specific stiffness have been widely applied in automobile, aerospace, and other fields [1,2,3,4]. Composite laminates are one of the most commonly used configuration types. However, the interlaminar properties of composite laminates are much lower than the in-plane properties because of the lack of reinforcement in the thickness direction. At the same time, owing to the mismatch of Poisson’s ratio between the laminates and the anisotropy of the coefficient of thermal expansion, serious stress concentration will be produced at locations with variable thickness, free edges, and hole edges. This weak interlaminar performance and high interlaminar stress can easily lead to interlaminar failure occurring at the interface between adjacent layers in the form of interlayer debonding, which is called delamination. Delamination is one of the most common and important failure modes in composite structures [5,6,7]. The occurrence and growth of delamination will lead to a significant reduction in the strength and stiffness of composite structures and may even cause unpredictable catastrophic failure [8,9,10]. This is extremely unfavorable to the integrity, life, and safety of the structures and will seriously restrict the application of composite materials in engineering structures. Therefore, the studies on delamination behavior are of great significance and practical value and are also the focus of damage tolerance design and analysis of composite structures [11,12].

Moreover, aerospace composite structures may be exposed to thermal conditions [13,14,15]. Resin matrix composites are very sensitive to high temperature. The variation in temperature will change the mechanical properties of components and the internal stress between composite layers and then affect the delamination propagation behavior of composite materials. In order to ensure the reliable application of carbon fiber-reinforced composites under service conditions, it is necessary to quantitatively characterize the temperature effect on the delamination behavior and failure mechanism of carbon fiber-reinforced composites. However, the importance of the degradation of the fracture toughness caused by the thermal environment has been ignored, based on the relatively small number of published studies, especially for the mode II delamination. More efforts should be made to study mode II delamination to promote the establishment of test standards.

Limited studies have shown that the relationship between the measured mode II fracture toughness and temperature is still not clear. Hashemi et al. [16] found that the mode II fracture toughness of AS4/PEEK laminates increased with the temperature increasing from 20 °C to 130 °C. A similar conclusion was also made by Machado et al. [17]. They measured the mode II fracture toughness of laminates fabricated by the carbon/epoxy composite (SEAL^®^ Texipreg HS 160 RM) at temperatures of 20 °C, 80 °C and −30 °C. Mode II fracture toughness of AS4/3501-6 was also found to increase with temperatures in the range of −50 °C to 100 °C [18], owing to increasing matrix ductility. However, Davies and Charentenay [19] found that there was no temperature effect on the mode II fracture toughness of T300/914C laminates evaluated in the range from 30 °C to 120 °C. More researchers found that the mode II fracture toughness decreased with the increase in temperature [20,21,22]. The results of Sjögren and Asp [23,24] showed that the mode II fracture toughness of HTA/6376C laminates decreased with the increase in temperature from −50 °C to 100 °C. However, the fracture surface characteristics at different temperatures were similar to each other. It was observed that the crack jumped between the upper and lower boundaries of the fiber when the temperature was 100 °C. However, at room temperature, the delamination grew along the upper boundary of the fiber. Cowley and Beaumont [25] performed end-notched flexure (ENF) tests on composite laminates fabricated by IM8/APC and IM8/954-2 materials. They found that the mode II fracture toughness decreased more obviously with the increase in temperature, especially when close to the glass transition temperature of the material. Davidson et al. [26] found that the matrix ductility increased with the increase in temperature, resulting in the decrease in mode II fracture toughness of T800H/3900-2 laminates. Boni et al. [15] measured the mode II fracture toughness of Hexcel 913C-HTA laminates evaluated at room temperature and 70 °C and found that it decreased at a higher temperature. Zulkifli and Azari [27] studied the mode II delamination growth behavior of silk fiber/epoxy laminates at 20 °C, 23 °C, 26 °C, 38 °C, 50 °C and 75 °C. It was shown that the sliding cracking of matrix and fiber appeared during the experiment, and the mode II fracture toughness at 75 °C showed a 71% decrease compared to that at room temperature.

The aerospace field has shown much interest in studying the effect of temperature on the mode II delamination behavior of widely used composites for design and verification purposes. To the authors’ knowledge, the mode II resistance curve (*R*-curve), which relates the strain energy release rate (SERR) to the delamination growth length, is still lacking in-depth investigation, especially in the thermal environment. This information is important and valuable if a damage tolerance philosophy is to be applied to the design of composite structures [28]. In addition, accurate simulation of the mode II delamination is required. Therefore, this study aims to investigate the temperature dependence of the mode II delamination behavior and establish a numerical framework for the delamination simulation based on the cohesive zone model.

## 2. Delamination Tests on the ENF Specimen

### 2.1. Specimen and Test Set-Up

Specimens were fabricated by carbon fiber T800/epoxy prepregs, which were provided by the Cytec Corporation. The layer-up panel was cured according to the supplier’s suggested procedures. The basic material properties are listed in Table 1, where *E*_1_, *E*_2_, and *E*_3_ are the Young’s moduli in the 1-direction (main orthotropic material axe), 2-direction (perpendicular orthotropic material axe) and 3-direction, respectively; *G*_12_, *G*_13_ and *G*_23_ are the shear moduli in the 1-2 plane, 1-3 plane, and 2-3 plane, respectively; *ν*_12_, *ν*_13_ and *ν*_23_ are the Poisson’s ratios in the 1-2 plane, 1-3 plane, and 2-3 plane, respectively. *X*_T_ and *X*_C_ are the tensile and compressive strengths in the 1-direction, respectively; *Y*_T_ and *Y*_C_ are the tensile and compressive strengths in the 2-direction, respectively. Z_T_ and *Z*_C_ are the tensile and compressive strengths in the 3-direction, respectively; *S*_12_, *S*_13_, and *S*_23_ are the shear strengths in the 1-2 plane, 1-3 plane, and 2-3 plane, respectively. The cured panel was cut into specimens with uniform geometrical dimensions of 180 mm length, 25 mm width and 4.8 mm thickness. A 40 mm long and 25 mm wide Teflon film was prearranged between the center plies to create an artificial pre-crack for the delamination. Unidirectional laminates with a stacking sequence of [0/0]_12_ were studied here.

The edges of specimens were painted with a thin layer of white correction fluid to facilitate the identification of the crack tip. The edge of the specimen was monitored by a traveling microscope, enabling continuous reading of the position of the crack tip with a precision of at least 0.1 mm. An insulated test chamber was used to keep the specimens at the designated temperature by electrical heating. Interior fans in the chamber were used to circulate the air and ensure a uniform temperature environment. The test was performed when the specimen remained at the stabilized temperature for about half an hour to allow the specimen to arrive at the equilibrium temperature. The chamber had one large, outfitted glass window and inside lighting, which enabled the observation of the position of the crack tip and the measurement of the delamination length during the tests.

### 2.2. Test Procedure

**Figure 1:**
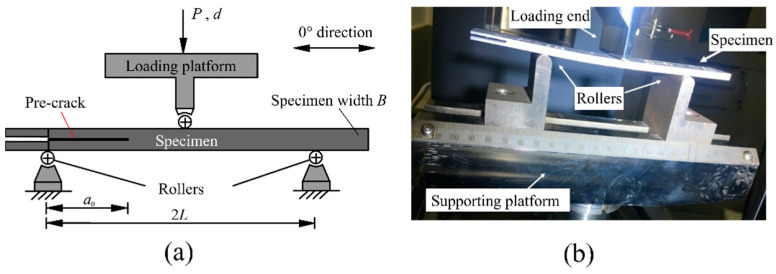
(**a**) Schematic diagram and (**b**) picture of the ENF test set-up.

Mode II delamination was achieved by the end-notched flexure (ENF) test. The ENF tests were conducted following the ASTM standard D7905/D7905M-14 [29] on a computer-controlled MTS 858 testing machine. Displacement controlling mode was adopted. The load was applied through a loading roller as shown in Figure 1. The total span between the external rollers was set at 100 mm. The ratio of the initial delamination length to the half span, *a*_0_/*L*, was set at 0.7 [30]. In this case, the delamination could be initiated in a relatively stable manner, followed by continued and relatively stable delamination growth over a relatively long distance before reaching the maximum load.

Before conducting the mode II delamination test, it was necessary to calibrate the specimen compliance in order to determine the relationship between the compliance *C* and the delamination length *a*. The derivative of the specimen compliance with respect to the delamination length *∂C*/*∂a* was used to calculate the mode II fracture toughness. By moving the position of the specimen on the support roller to achieve different delamination lengths, the compliance calibration was applied to obtain five data points. In order to facilitate the compliance calibration and positioning, the specimen edges were marked at 15, 20, 25, 30, and 35 mm away from the end of the pre-crack. At the same time, a ruler was set on the support platform, and the center line of the support roller was marked for accurate placement of the specimen. When placing the specimen, the marking line on the roller was aligned with the scale line on the ruler of the support platform to ensure an exact delamination length during the compliance calibration and the delamination test. According to ASTM standard D7905/D7905M-14 [29], the maximum force during the compliance calibration should be less than 50% of the ultimate load. In this work, the load applied during the compliance calibration was in the linear elastic stage of the material. The maximum applied load was 800 N and then unloaded. After each compliance test, the specimen was fully unloaded, and then the procedure of “adjusting the distance-holding heating-reloading-unloading” was repeated until the compliance data corresponding to five different delamination lengths were obtained. The loading speed during delamination tests was 0.1 mm/min in order to obtain slow delamination growth and maximize the number of data points obtained. The unloading speed was 0.5 mm/min. It should be noted that once the incubator was opened to adjust the delamination length, re-heating was required for more than 10 min to ensure the stability of the tested temperature.

### 2.3. Date Reduction Method

The mode II fracture toughness can be determined by linear elastic fracture mechanics. Three different calculation methods are available [31], including the Experimental Compliance Method (ECM) [29], the Simple Beam Theory Method (SBT), and the Corrected Beam Theory with Effective Crack Length Method (CBTE) [32]. All of them can be expressed as the classical Irwin–Kies equation:

(1) $$G_{\mathrm{II}}=\frac{P^{2}}{2b}\frac{\partial C}{\partial a}$$

where *P* is the applied load, *C* is the specimen compliance, *a* and *b* are the delamination length and the specimen width, respectively.

#### 2.3.1. ECM Method

The ECM is established based on the assumption of a cubic relationship between the specimen compliance and the delamination length:

(2) $$C=A+m\cdot a^{3}$$

where *C* is the specimen compliance (the ratio between the displacement and the load, *δ*/*P*), the parameters *m* and *A* are the slope and intercept, respectively, of the fitting curve obtained by the least squares linear regression analysis. Substituting Equation (2) into Equation (1), the expression for mode II fracture toughness can be derived.

(3) $$G_{\mathrm{II}}=\frac{3P^{2}a^{2}m}{2b}$$

#### 2.3.2. SBT Method

According to the Simple Beam Theory, the compliance of the ENF specimen is as follows.

(4) $$C=\frac{\delta }{P}=\frac{3a^{3}+2L^{3}}{8bh^{3}E_{1}}$$

Substituting Equation (4) into Equation (1), the expression for the fracture toughness is:

(5) $$G_{\mathrm{II}}=\frac{9P^{2}a^{2}}{16b^{2}h^{3}E_{1}}$$

where *L* is the half span, *δ* is the applied displacement, *P* is the applied load, *a* is the delamination length, *b* is the specimen width, *h* is a half of the specimen thickness, *E*_1_ is the flexural modulus. The limitation of the SBT method is that the contribution of shear deformation of the specimen to the energy release rate is neglected. Zero compliance at the crack root is assumed in Equation (4). However, in an actual case, there is some deflection and rotation at the crack tip.

#### 2.3.3. CBTE

The above ECM and SBT methods must visually observe the position of the crack tip, which is usually not easy to achieve in the mode II delamination. This is because there is no clear opening during the delamination growth [33]. Even with the help of a microscope with high magnification, it is difficult to distinguish between the damage and the delamination growth [34]. Therefore, the CBTE method without the requirement for visual observation of the crack tip is proposed. The compliance of the ENF specimen is defined as Equation (6).

(6) $$C=\frac{\delta }{P}=\frac{3{\left(a_{e}\right)}^{3}+2L^{3}}{8bh^{3}E_{1}}$$

Substituting Equation (6) into Equation (1), the equation for the fracture toughness is given by:

(7) $$G_{\mathrm{II}}=\frac{9P^{2}a_{e}{}^{2}}{16b^{2}h^{3}E_{1}}$$

where *a_e_* is the effective delamination length and can be obtained by Equation (8).

(8) $$a_{e}={\left(\frac{1}{3}(8bCh^{3}E_{1}-2L^{3})\right)}^{\frac{1}{3}}$$

## 3. Test Results

### 3.1. Compliance Calibration Results

**Figure 2:**
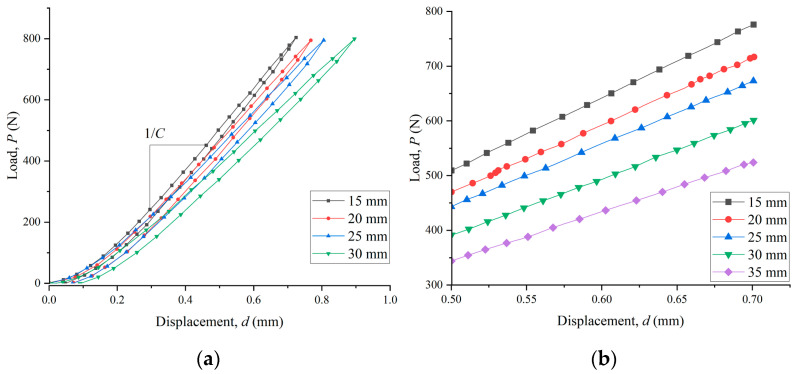
*P*-*d* curves from the compliance calibration of ENF specimens (**a**) original curve and (**b**) linear elastic section for the determination of compliance.

**Figure 3:**
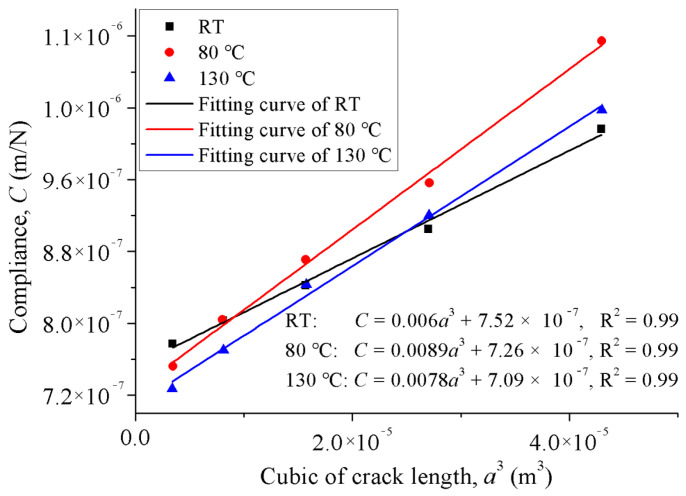
Compliance calibration of ENF specimens evaluated at different temperatures.

For the ECM method, the load–displacement curves of specimens with different delamination lengths were used to obtain the relationship between the compliance and the crack length. Figure 2a shows the obtained load–displacement curve of the specimen in the compliance calibration process. As shown in Figure 2b, the linear sections of the curves are abstracted for linear fitting in order to determine the specimen compliance. In this way, the plots of the compliance against the cubic of crack length at RT, 80 °C, and 130 °C are presented in Figure 3, which are fitted by Equation (2). For all temperatures, it can be seen that a good linear relation (with the R^2^ coefficient equal to 0.99) was obtained between the compliance and the cubic of crack length. The detailed fitting formulas are given in Figure 3. The values of the *m* at RT, 80 °C, and 130 °C were 0.006, 0.0089, and 0.0078, respectively. These values were used in Equation (3) for the calculation of fracture toughness.

### 3.2. Load–Displacement Curve

**Figure 4:**
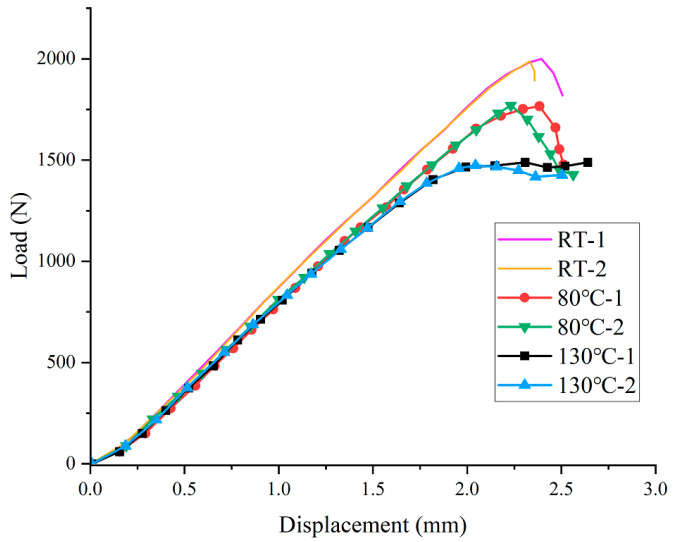
Load–displacement curves of ENF specimens evaluated at different temperatures.

Figure 4 shows the load–displacement curves of the ENF specimens under various temperature conditions. From this figure, it can be seen that all specimens exhibit a short nonlinear behavior at the initial stage of loading when the specimen first contacted with the loading nose. Then, the load–displacement curve exhibits a linear part, and the load gradually increases until reaching the maximum value. Before arriving at the final load drop, the curve exhibits a certain degree of non-linear response owing to the formation of a process zone of the matrix deformation and micro cracks at the crack tip, and delamination failure. Subsequently, the load decreased with further delamination growth. Comparing the load–displacement curves at different temperatures, both the specimen stiffness and the maximum load decreased with the increase in temperature. At room temperature, the load dropped rapidly after reaching the maximum value, which illustrated the relatively unstable delamination growth. However, at high temperatures, the load drop process tended to be gentler, especially when the temperature was 130 °C. More significant nonlinear behavior appeared, and the specimen carried the load in a relatively stable manner. This phenomenon was consistent with the experimental results in [35].

### 3.3. Fracture Toughness

**Figure 5:**
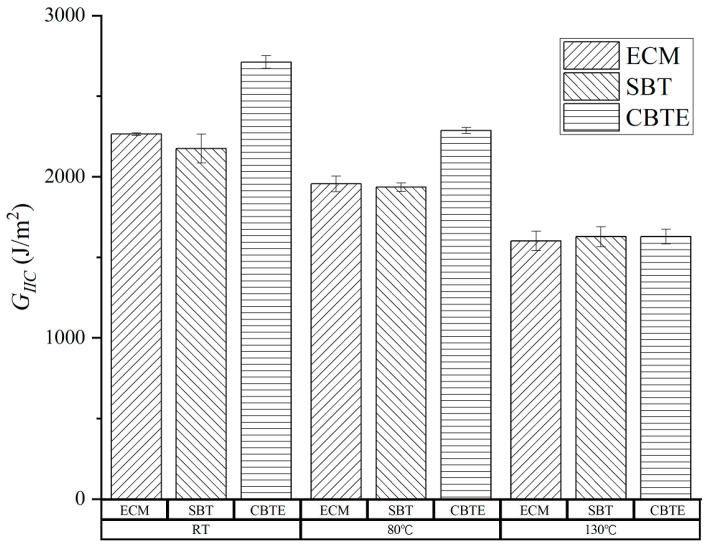
Comparison of mode II fracture toughness calculated by different data reduction methods.

Based on the recorded load, deflection, and delamination length, the mode II fracture toughness under various temperature conditions was calculated using the three data reduction methods given in Section 2.3. The obtained results are shown in Figure 5. It can be seen that the ECM and SBT produced similar results. However, At RT and 80 °C, the fracture toughness values obtained by the CBTE method were obviously higher than those of the other two methods. Only at 130 °C was there no obvious difference between the results calculated by the three methods. Detailed values of the mode II fracture toughness are listed in Table 2. The mean value and standard deviation are also listed in Table 2. In this work, the ENF tests were conducted referring to the ASTM standard D7905/D7905M-14 and the ECM is the only one method covered in this ASTM standard. Therefore, the mode II fracture toughness calculated by the ECM method ECM was compared. It was determined that the fracture toughness decreased with the increase in temperature. The fracture toughness values at 80 °C and 130 °C decreased by 13.88% and 29.47%, respectively, compared with that at room temperature. With the increase in temperature, the decrease in fracture toughness may be caused by the degradation of the matrix, which led to the decrease in shear strength and stiffness and the increase in resin ductility [22,36,37].

**Figure 6:**
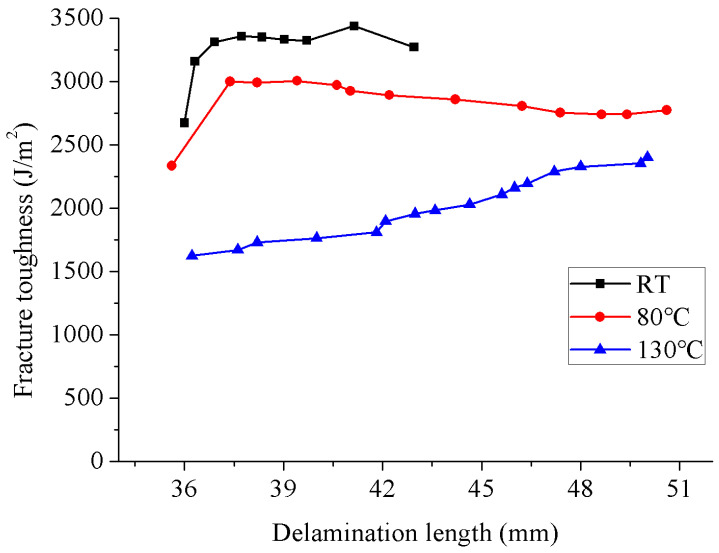
*R*-curves of ENF specimens evaluated at different temperatures.

An obvious *R*-curve was observed over the entire evaluated temperature range, and the *R*-curves are shown in Figure 6. The *R*-curve behavior was also presented in mode I delamination [38,39] and mixed-mode I/II delamination [40,41,42] of composite laminates. Wang et al. [38,39] carried out mode I delamination tests on six kinds of T800/X850 laminates with different interfaces of 0°/0°, +22.5°/−22.5°, +45°/−45°, 90°/90°, 0°/45°, and 0°/90°. An interface-dependent analytical model was proposed for the plateau fracture toughness. Gong et al. [40] experimentally determined the mixed-mode I/II *R*-curves of the unidirectional and multidirectional laminates made from carbon/epoxy composites by using the mixed-mode bending tests. It was found that the initial and steady-state fracture toughness values were strongly influenced by the interfacial ply and the mode mixity. The fiber bridging length of mode I delamination was much higher than that of mode II delamination. However, the fiber bridging length in the mixed-mode I/II delamination was approximately the same for unidirectional laminates and multidirectional laminates with a +22.5°/−22.5° interface. Furthermore, mixed-mode I/II delamination tests were performed on multidirectional T700/QY9511 laminates with a +45°/−45° interface [41,42]. Mixed-mode I/II *R*-curves were obtained, while the fracture toughness increased with the increase in delamination growth length while there was no plateau value. They also found a similar *R*-curve behavior of fatigue delamination resistance, which is also mainly resulted by the bridging fibers [43,44,45,46,47]. However, the resulting damage status in fatigue delamination is usually different from that in static delamination. For the mode II delamination studied here, the *R*-curve primarily arose from an increasing amount of crack-tip plasticity developing as the crack grew [16]. At RT and 80 °C, the fracture toughness gradually increased with the delamination growth until reaching a relatively stable value after a certain delamination growth length. At 130 °C, the fracture toughness linearly increased with the delamination growth, and no stable value was exhibited. With the increase in temperature, a longer delamination growth length is required to achieve the saturation state of the formed crack tip plasticity. However, because of the limitation of the effective crack length in the ENF test, the fracture toughness corresponding to the delamination length higher than 50 mm could not be obtained.

### 3.4. Analysis on the Fracture Surface

**Figure 7:**
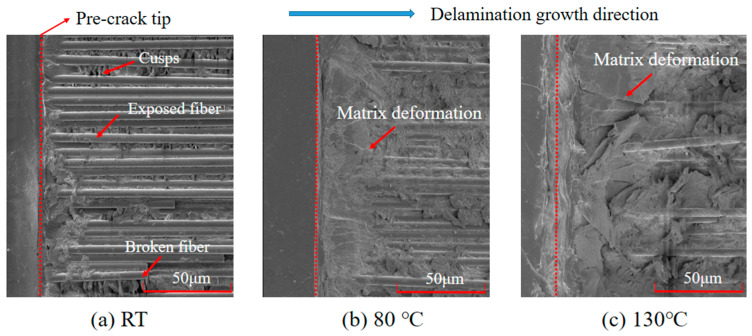
SEM images of fracture surfaces from the specimens tested at different temperatures.

A fractographic study was conducted on the ENF specimens in order to analyze the failure mechanisms at different temperatures. The tested specimens were opened and the exposed fracture surfaces were gold sputter-coated and then examined with a scanning electron microscope (SEM) at a magnification of 2000. It was noted that the main micro characteristics of the fracture surfaces were shear cusps or hackles. At room temperature, a brittle failure mode with relatively little matrix deformation was observed on the fracture surface. The low fiber/matrix debonding strength resulted in a large amount of exposed fibers, as shown in Figure 7a. For the delamination tests at high temperatures, an increase in the resin ductility was observed with the increase in temperature, a feature that was highlighted by the higher degree of matrix deformation and the amount of matrix adhered to the fibers on the fracture surfaces, as shown in Figure 7b,c. The decrease in the amount of unstable delamination growth from room temperature to the elevated high temperature also reflected this transition from brittle to ductile behavior. This was consistent with the discussion in Section 3.3.

## 4. Numerical Simulation for the Mode II Delamination in ENF Specimens

In this section, a numerical framework is established for the mode II delamination growth behavior at the thermal conditions. Influences of temperature on the basic material mechanical parameters were considered using a material model, which can quantitatively characterize the temperature-dependent effect on the mechanical properties of composite materials based on exponential functions of the temperature. The delamination growth behavior was modeled using the cohesive elements with a bilinear constitutive law. The effects of interfacial parameters on the simulations were investigated, and a suitable value set for the interfacial parameters was determined.

### 4.1. A model for the Thermal-Affected Material Properties

The constitutive law of a transverse isotropic material can be expressed as ***ε*** = ***S***•***σ***, where ***ε***, ***S***, and ***σ*** are the matrixes of engineering strain, compliance, and stress, respectively. The element *S_ij_* is a function of engineering constants *E_ij_*, *ν_ij_*, *G_ij_* (*i*, *j* = 1, 2, 3) of the composite material [48]. In order to include the temperature effect on the material mechanical properties, the compliance matrix ***S*** is modified, as shown in Equation (9):

(9) $$S^{\mathrm{temp}}=\left[\begin{array}{cccccc} 1/E_{11}^{\mathrm{temp}} & -\nu_{21}^{\mathrm{temp}}/E_{22}^{\mathrm{temp}} & -\nu_{31}^{\mathrm{temp}}/E_{33}^{\mathrm{temp}} & 0 & 0 & 0\\ & 1/E_{22}^{\mathrm{temp}} & -\nu_{32}^{\mathrm{temp}}/E_{33}^{\mathrm{temp}} & 0 & 0 & 0\\ & & 1/E_{33}^{\mathrm{temp}} & 0 & 0 & 0\\ & & & 1/G_{23}^{\mathrm{temp}} & 0 & 0\\ & & & & 1/G_{31}^{\mathrm{temp}} & 0\\ \mathrm{symm}. & & & & & 1/G_{12}^{\mathrm{temp}} \end{array}\right]$$

where “temp” denotes the thermal condition. A non-dimensional temperature T* as Equation (10) is introduced to evaluate the temperature-dependent characteristics of the composite material, which has also been adopted by other researchers [49,50].

(10) $$T^{*}=\frac{T_{g}-T_{\mathrm{opr}}}{T_{g}-T_{\mathrm{rm}}}$$

Here, *T*_g_ is the glass transition temperature, *T*_opr_ is the operation temperature, and *T*_rm_ is the room temperature. A united model [51], such as Equation (11), can be used to quantitatively characterize the temperature-dependent effect on the mechanical property of composite materials. The matrix and fiber stiffness and strength data are empirically fitted by exponential formulas as a function of T*. This model was first proposed for the hygrothermal environment. When there is no moisture absorption, this model degrades to the case with only temperature effect and is also applicable.

(11) $$\begin{array}{l} \frac{E_{11}^{\mathrm{temp}}}{E_{11}^{0}}={\left(T^{*}\right)}^{a},\frac{E_{22}^{\mathrm{temp}}}{E_{22}^{0}}={\left(T^{*}\right)}^{b},\frac{G_{12}^{\mathrm{temp}}}{G_{12}^{0}}={\left(T^{*}\right)}^{c}\\ \frac{X_{T}^{\mathrm{temp}}}{X_{T}^{0}}={\left(T^{*}\right)}^{d},\frac{X_{C}^{\mathrm{temp}}}{X_{C}^{0}}={\left(T^{*}\right)}^{e},\frac{Y_{T}^{\mathrm{temp}}}{Y_{T}^{0}}={\left(T^{*}\right)}^{f}\\ \frac{Y_{C}^{\mathrm{temp}}}{Y_{C}^{0}}={\left(T^{*}\right)}^{g},\frac{S_{12}^{\mathrm{temp}}}{S_{12}^{0}}={\left(T^{*}\right)}^{h},\frac{\nu_{12}^{\mathrm{temp}}}{\nu_{12}^{0}}=1 \end{array}$$

Here, the symbols *a*–*h* denote empirical constants. *X*_T_, *X*_C_, *Y*_T_, *Y*_C_ and *S*_12_ are the longitudinal tensile and compressive, transverse tensile and compressive, and in-planar shear strengths, respectively. The superscript “0” denotes the room temperature. Values of the exponents in Equation (11) can be determined by a micro-level based method [50,52,53]. As shown in Equation (12), mechanical properties *E*_33_, *G*_13_, *ν*_13_, *G*_23_, *Z*_T_, *Z*_C_ and *S*_13_ at the thermal condition are determined based on the transverse isotropic assumption. The *ν*_23_ and *S*_23_ are determined based on the method proposed by Christensen [54] and Pinho et al. [55]. The values of the exponents in Equation (11) are listed in Table 3 from which the basic mechanical properties of the composite material at 80 °C and 130 °C can be determined and are also listed in Table 3.

(12) $$\begin{array}{l} E_{33}^{\mathrm{temp}}=E_{22}^{\mathrm{temp}},G_{13}^{\mathrm{temp}}=G_{12}^{\mathrm{temp}},\nu_{13}^{\mathrm{temp}}=\nu_{12}^{\mathrm{temp}},\\ \nu_{23}^{\mathrm{temp}}=\nu_{12}^{\mathrm{temp}}\left(1-\nu_{12}^{\mathrm{temp}}E_{22}^{\mathrm{temp}}/E_{11}^{\mathrm{temp}}\right)/\left(1-\nu_{12}^{\mathrm{temp}}\right),\\ G_{23}^{\mathrm{temp}}=E_{22}^{\mathrm{temp}}/2\left(1+\nu_{23}^{\mathrm{temp}}\right),\\ Z_{T}^{\mathrm{temp}}=Y_{T}^{\mathrm{temp}},Z_{C}^{\mathrm{temp}}=Y_{C}^{\mathrm{temp}},S_{13}^{\mathrm{temp}}=S_{12}^{\mathrm{temp}},\\ S_{23}^{\mathrm{temp}}=\frac{Y_{C}^{\mathrm{temp}}}{2\tan(53^{\circ })}. \end{array}$$

### 4.2. Cohesive Zone Model with a Bilinear Constitutive Law

**Figure 8:**
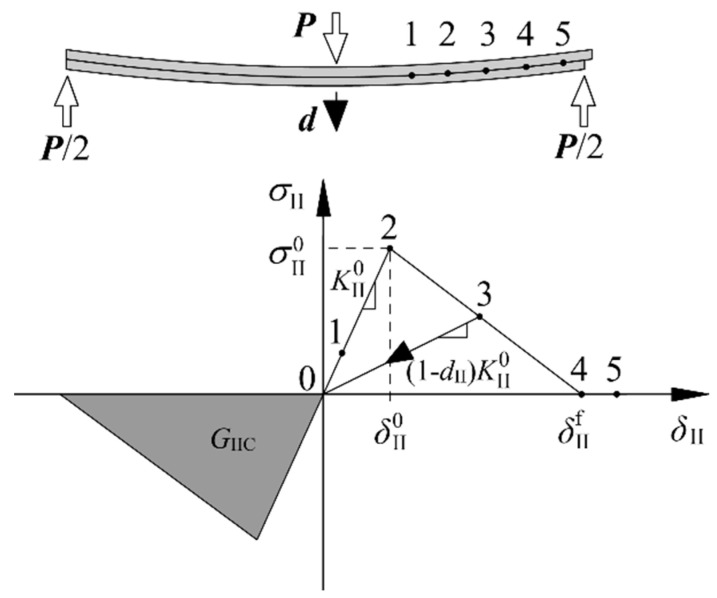
Bilinear constitutive law for the mode II delamination in an ENF specimen.

Different methods have been proposed to simulate the delamination growth behavior, such as the virtual fracture closure technique (VCCT) [56], extended finite element method (XFEM) [57,58], and cohesive zone model (CZM) [7,59]. The XFEM can effectively model the delamination migration [60,61] in composite structures [62]. In addition, Reddy and co-workers [63,64] illustrated that delamination can be conveniently modeled using an element deletion/failure method in conjunction with a non-local fracture criterion. They developed a novel three-dimensional, rate form-based finite-deformation constitutive theory to describe the damage and fracture in viscoelastic solids. The CZM is a prevailing method which combines strength-based analysis to predict delamination initiation and fracture mechanics to predict delamination growth. The constitutive law relates cohesive traction (*σ*) to crack opening/shearing displacement (*δ*) at the interface. Different constitutive laws have been proposed for different material systems and structural forms, such as bilinear, exponential, trapezoidal, and multi-linear [65]. To deal with the effect of fiber bridging on the delamination behavior, modified delamination growth criteria and modified cohesive zone model were proposed. In the modified delamination growth criteria, the *R*-curve was integrated into the traditional cohesive zone model with bilinear constitutive law [41,42]. Gong et al. [65] proposed a physical-based three-linear CZM superposed by two bilinear CZMs that represent two different phenomena including the quasi-brittle matrix fracture characterized by a higher peak stress and a shorter relative opening displacement, and the fiber bridging characterized by a lower peak stress and a longer relative opening displacement. Furthermore, a novel four-linear cohesive law was developed [66], which was established based on the realistic failure mechanism during the delamination growth. All the necessary cohesive parameters can be obtained by tests except for the fracture toughness ratio, which is introduced to characterize the non-linear softening behavior of the bridging fibers in a simple way. In addition, the implementation of the four-linear cohesive law in FEM is easier and more efficient than the non-linear. In order to determine the bridging law that can be applied in the multi-linear cohesive law, different semi-analytical methods have been developed [67,68,69] based on the beam theory. Only experimental load and displacement data are required and the visual observation for the delamination is avoided, which makes the semi-analytical methods time saving and cost effective. A simple procedure was also established for determining the bridging law [70]. The crack opening displacement at the initial pre-crack tip was obtained by a numerical method. The bridging law was determined via the *J*-integral method. The obtained results agreed well with the experimental ones. In this study, the bilinear cohesive law, which is the most widely used because of its simplicity and intuitive physical meaning [71], was adopted. Figure 8 shows the constitutive behavior of pure mode II delamination in an ENF specimen.

Interface stiffness $K_{\mathrm{II}}^{0}$ is used to characterize the mechanical behavior of the cohesive element in the linear elastic range (point 1 in Figure 8). When the interface stress reaches the shear strength $\sigma_{\mathrm{II}}^{0}$ (point 2), the damage onset in cohesive elements occurs. The relative displacement at this point can be expressed as: $\delta_{\mathrm{II}}^{0}=\sigma_{\mathrm{II}}^{0}/K_{\mathrm{II}}^{0}$. Point 3 indicates that the element is in the softening stage and has been partially damaged. The interface stiffness is degraded to *K*_II_ = (1 − *d*_II_) $K_{\mathrm{II}}^{0}$, where *d*_II_ ∈ [0,1] is the damage variable. The element completely fails and loses its load-bearing capacity when the interface stiffness reduces to zero (point 4). According to Griffith’s fracture theory, the area under the traction-relative displacement relationship is equal to the fracture toughness *G*_IIC_ as shown in Equation (13):

(13) $$\int_{0}^{\delta_{\mathrm{II}}^{f}}\sigma_{\mathrm{II}}d\delta_{\mathrm{II}}=G_{\mathrm{IIC}}$$

where $\delta_{\mathrm{II}}^{f}$ is the relative displacement when the cohesive element just loses the load-bearing capacity. The detailed constitutive law is as follows:

(14) $$\sigma_{\mathrm{II}}=\left\{ \begin{array}{ll} K_{\mathrm{II}}\delta_{\mathrm{II}}, & \delta_{\mathrm{II}}^{\max}\leq \delta_{\mathrm{II}}^{0}\\ \left(1-d_{\mathrm{II}}\right)K_{\mathrm{II}}\delta_{\mathrm{II}}, & \delta_{\mathrm{II}}^{0}<\delta_{\mathrm{II}}^{\max}\leq \delta_{\mathrm{II}}^{f}\\ 0, & \delta_{\mathrm{II}}^{\max}>\delta_{\mathrm{II}}^{f} \end{array}\right.,$$

(15) $$d_{\mathrm{II}}=\frac{\delta_{\mathrm{II}}^{f}\left(\delta_{\mathrm{II}}^{\max}-\delta_{\mathrm{II}}^{0}\right)}{\delta_{\mathrm{II}}^{\max}\left(\delta_{\mathrm{II}}^{f}-\delta_{\mathrm{II}}^{0}\right)},\;d_{\mathrm{II}}\in \left[0,1\right]$$

where $\delta_{\mathrm{II}}^{\max}$ is the maximum relative sliding displacement and is defined as:

(16) $$\delta_{\mathrm{II}}^{\max}=\max\left\{\delta_{\mathrm{II}}^{\max},\left|\delta_{\mathrm{II}}\right|\right\}.$$

### 4.3. Numerical Model of the ENF Specimen

**Figure 9:**
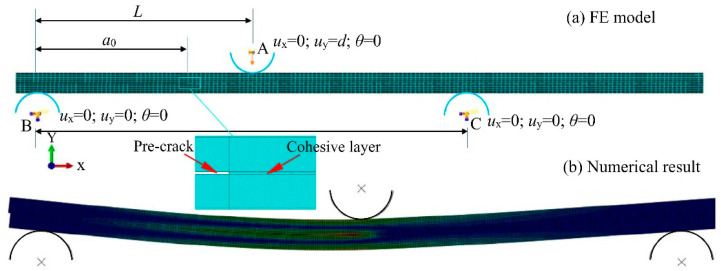
Simulation of the ENF specimen (**a**) FE model and (**b**) numerical result.

Considering the transverse isotropy of the unidirectional laminate studied here, a two-dimensional finite element model of the ENF specimen was established in the Abaqus commercial FE software, as shown in Figure 9. A low computational cost was achieved without reducing the prediction accuracy, as shown in the following simulation. The specimen was modeled as the upper and lower arms tied with a cohesive layer in the uncracked region, while contact interactions were present in the pre-cracked region to prevent the penetration between the arm surfaces. The two arms were meshed with four-node plane stress elements with a coarse mesh along the specimen length. Four-node two-dimensional cohesive elements (COH2D4) were pre-arranged along the middle plane of the specimen to simulate the delamination growth [72]. In the thickness direction, one element for each composite layer was used. A refined mesh was adopted for the cohesive layer to capture accurate stress/strain distributions in the cohesive zone and ensure the independence of simulation results on the mesh size. To reproduce the ENF test, the specimen was sustained by two semi-cylindrical rigid supports (B and C) with a span of 100 mm and loaded by a central nose (A). Boundary conditions were assigned to the two supports through their reference points, which were constrained in translation and rotation. A displacement boundary condition was applied to the central semi-cylinder A, and the horizontal translation was inhibited, such as the in-plane rotation. In this work, the delamination growth was evaluated by comparing the SERR of the cohesive element with the fracture toughness. The fracture toughness was obtained from the tests as presented in Section 3.3. The critical interface parameters of the cohesive element were numerically determined, as shown in the following, Section 4.4.

### 4.4. Simulated Results

When using the cohesive zone model for the delamination modeling, the interface stiffness, the viscosity coefficient of the cohesive elements, the mesh size, and the interface strength are critical parameters. The interface stiffness is a non-physical parameter used to maintain the rigid connection between the undamaged cohesive elements and the laminates. Ideally, the interface stiffness should be infinite, so that the overall stiffness of the structure is accurately modeled. However, it will result in a converging problem and spurious oscillation. Therefore, a reasonable value of the interface stiffness should be adopted. The viscosity is introduced in the cohesive elements in order to improve the convergence of calculation. The viscous regularization taken by the constitutive law is realized by permitting the stresses beyond the interface strength, and it adopts a relatively small characteristic time increment. Computational costs and influences on predictions should be considered when determining the optimal viscosity coefficient. Reasonable mesh size of the cohesive element is particularly important for the accuracy of simulation results. The selection of mesh size depends on the length of the cohesive zone and the number of cohesive elements in this zone. The length of the cohesive zone is related to the interface strength [73]. In order to accurately capture the stress/strain field in the cohesive zone, a sufficiently fine meshing for the cohesive elements is required. At present, there is still no universal rule for determining the values of those parameters. In this work, the following procedure was adopted based on the trial-and-error method. First, the mesh of the cohesive element was fine to ensure enough elements in the cohesive zone, and the effect of the interface stiffness on the elastic stage of the load–displacement response was investigated. Then, the effect of the viscosity coefficient of cohesive elements was studied. Based on the determined values of interface stiffness and viscosity coefficient, the influence of mesh size and interface strength was finally studied. From this procedure, the suitable value set of these critical parameters were obtained.

**Figure 10:**
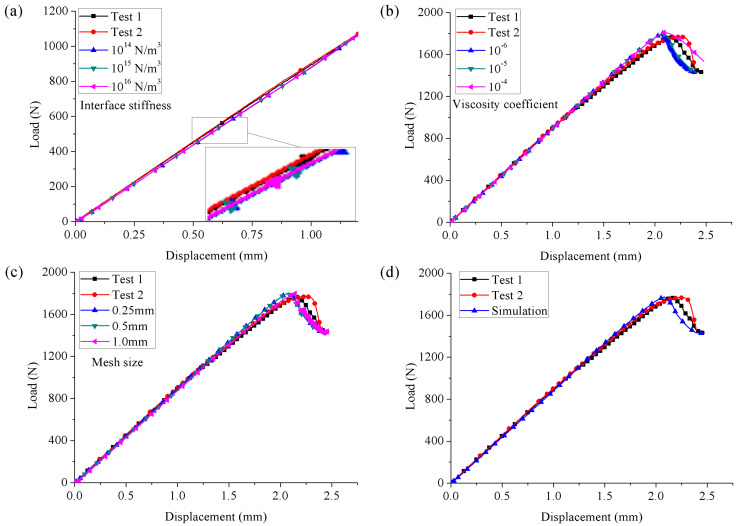
Influence of (**a**) interface stiffness, (**b**) viscosity coefficient, (**c**) mesh size on the predicted load–displacement response and (**d**) numerical results of the ENF test at 80 °C.

Three kinds of interfacial stiffness (10^14^ N/m^3^, 10^15^ N/m^3^ and 10^16^ N/m^3^) were studied to reveal its influence on the initial slope of the predicted load–displacement curve. The predictions are shown in Figure 10a. ‘Test 1’ and ‘Test 2’ represented the experimental results of two tested specimens. For better comparisons, the initial nonlinear stage of the experimental results was removed. It indicated that the influence of interface stiffness was trivial and the predictions had good agreement with the experimental ones. Considering that a larger value of interface stiffness can ensure less structural stiffness loss, its value was reasonably chosen as 10^15^ N/m^3^ for the specimens evaluated at 80 °C. The influence of the viscosity coefficient (10^−6^, 10^−5^, and 10^−4^) on the predicted load–displacement curves is shown in Figure 10b and compared with the experimental ones. It can be seen that the viscosity coefficient had no effect on the linear and elastic stage, while it had an obvious effect on the subsequent load-drop stage. Consistent results were obtained when the viscosity coefficient was chosen as 10^−6^ and 10^−5^ because a higher viscosity coefficient usually results in a better convergence, which means fewer analysis steps and less computational time are required. Thus, the viscosity coefficient was chosen as 10^−5^ for the specimens evaluated at 80 °C. The predicted load–displacement curves with different mesh sizes (0.25, 0.5, and 1 mm) are shown in Figure 10c. It can be seen that the effect of the studied mesh size on the predictions was also trivial. Because a finer mesh size will increase the computational cost, the mesh size was chosen as 0.5 mm. The final numerical results are compared with experimental results in Figure 10d, and good agreement was achieved between them.

**Figure 11:**
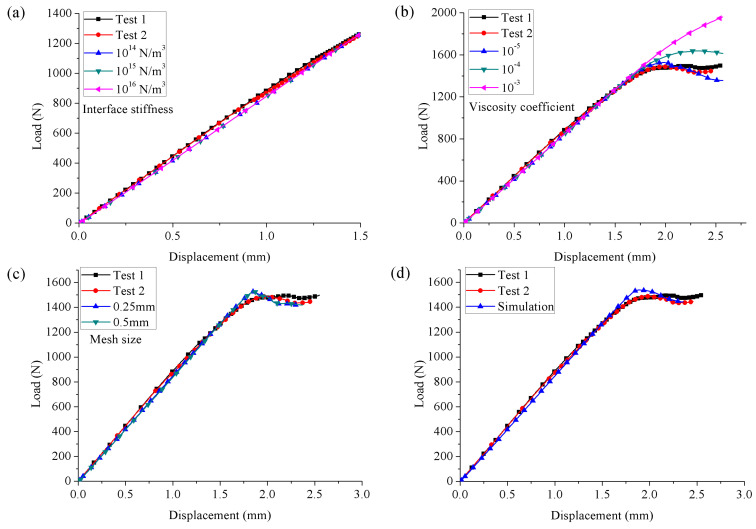
Influence of (**a**) interface stiffness, (**b**) viscosity coefficient, (**c**) mesh size on the predicted load–displacement response, and (**d**) numerical results of the ENF test at 130 °C.

The reasonable interfacial parameters for modeling the ENF test at 130 °C were determined by following the above method. Figure 11 shows the effects of interface stiffness (10^14^, 10^15^, and 10^16^ N/m^3^), viscosity coefficient (10^−5^, 10^−4^ and 10^−3^) and mesh size (0.25 and 0.5 mm) on the numerical results. It can be seen that in the studied range, the interface stiffness and mesh size did not affect the predictions. For different viscosity coefficients, the predicted linear stages were the same. However, only when its value was 10^−5^ was a satisfactory agreement between the predictions and the experimental results obtained for the load-drop stage. Therefore, the interfacial parameters of the cohesive elements suitable for ENF tests evaluated at 130 °C were as follows: interface stiffness of 10^15^ N/m^3^, viscosity coefficient of 10^−5^, and mesh size of 0.5 mm. Based on the suitable interfacial parameter set, the numerical model for simulating the mode II delamination of composite laminates was established. The final numerical results were obtained and are presented in Figure 11d, and they agreed well with the experimental results.

## 5. Conclusions

Mode II delamination tests were conducted on CFRP laminates using the ENF set-up to investigate the temperature dependence of delamination behavior. The fracture toughness at 80 °C and 130 °C decreased by 13.88% and 29.47%, respectively, compared with that at room temperature. This observation was consistent with most published results. The decrease in fracture toughness with the increase in temperature may be caused by the degradation of matrix, which led to the decrease in shear strength and stiffness and the increase in resin ductility. In addition, obvious *R*-curve behaviors were experimentally observed in the specimens tested at different temperatures, which suggested that the fracture toughness increased with the increase in temperature. The *R*-curve was mainly caused by the increasing amount of crack-tip plasticity. At RT and 80 °C, the fracture toughness value was relatively stable after a certain delamination growth length. However, no obvious stable value was observed for the specimen tested at 130 °C, owing to the limitation of the effective crack length in the work. It may need a longer delamination growth length to achieve the stable value, and this requires further validation from future work. Micrographs of the fracture surfaces obtained via scanning electron microscope showed that a brittle failure mode with relatively little matrix deformation was observed in specimens evaluated at room temperature. However, with the increase in temperature, an increase in the matrix ductility was observed, as confirmed by the higher degree of matrix deformation and the amount of matrix adhered to the fibers on the fracture surfaces. The decrease in the amount of unstable delamination growth from the room temperature to the higher temperature also reflected this transition from brittle to ductile behavior.

Based on the cohesive zone model with a bilinear constitutive law, a numerical framework was established for the delamination simulation of ENF tests. Temperature effects on the mechanical parameters of the composite material, including the elastic and strength parameters, were considered. Detailed values of these mechanical parameters were determined by a power exponential model formulated as a function of a non-dimensional temperature. The basic elastic parameters and strengths decreased with the increase in temperature. The effects of critical cohesive parameters, including interface stiffness, viscosity, and mesh size, were numerically investigated. A suitable value set for the critical cohesive parameters was identified and applied to the delamination modeling. It indicated that the interfacial parameters of the cohesive elements suitable for ENF tests evaluated at 80 °C and 130 °C were as follows: interface stiffness of 10^15^ N/m^3^, viscosity coefficient of 10^−5^, and mesh size of 0.5 mm. The temperature did not seem to affect the suitable value of the interfacial parameter. The predicted load–displacement responses showed satisfactory agreement with the experimental, which illustrated the applicability and accuracy of the established numerical model. It is worthwhile to point out that the aerospace composite structures are usually exposed to thermal cycles. Further studies are required on the delamination behavior of those structures exposed to thermal cycles, which should be an interesting topic.

## Figures and Tables

**Table 1:** Basic mechanical properties of T800/epoxy material.

*E*_1_ (Gpa)	*E*_2_, *E*_3_ (Gpa)	*G*_12_, *G*_13_ (Gpa)	*G*_23_ (Gpa)	*ν*_12_, *ν*_13_	*ν*_23_
163.5	9.0	4.14	3.08	0.32	0.46
*X*_T_ (MPa)	*X*_C_ (MPa)	*Y*_T_, *Z*_T_ (MPa)	*Y*_C_, *Z*_C_ (MPa)	*S*_12_, *S*_13_ (MPa)	*S*_23_ (MPa)
2992	1183	70.6	278	172	105

**Table 2:** Mode II fracture toughness of ENF specimens evaluated at different temperatures. (Unit: J/m^2^).

**Temperature**	**Method**	**Test 1**	**Test 2**	**Mean Value**	**S.D.**	**CoV (%)**
RT	ECM	2269.68	2259.09	2264.39	5.29	0.2
	SBT	2237.93	2110.97	2174.45	63.48	2.9
	CBTE	2738.67	2684.17	2711.42	27.25	1.0
80 °C	ECM	1920.93	1989.62	1955.28	34.34	1.8
	SBT	1916.81	1953.56	1935.19	18.38	0.9
	CBTE	2273.08	2300.07	2286.58	13.50	0.6
130 °C	ECM	1644.45	1558.94	1601.70	42.78	2.7
	SBT	1584.24	1671.48	1627.86	43.63	2.7
	CBTE	1660.58	1595.40	1627.99	32.59	2.0

**Table 3:** Values of the exponents for the engineering constant calculations and the determined mechanical properties of T800/epoxy composites at high temperatures.

Item	*a*	*b*	*c*	*d*	*e*
Value	0.04	0.5	0.5	0.04	0.54
Item	*f*	*g*	*h*	*T*_g_/°C	*T*_rm_/°C
Value	0.50	0.50	0.50	185	23
Elastic property	80 °C	130 °C	Strength (MPa)	80 °C	130 °C
*E*_1_ (GPa)	160.7	156.6	*X*_T_	2941	2866
*E*_2_, *E*_3_ (GPa)	7.245	5.24	*X*_C_	936	661
*G*_12_, *G*_13_ (GPa)	3.33	2.41	*Y*_T_, *Z*_T_	56.8	41.2
*G*_23_ (GPa)	2.44	1.76	*Y*_C_, *Z*_C_	224	162
*ν*_12_, *ν*_13_	0.33	0.33	*S*_12_, *S*_13_	138	100
*ν*_23_	0.485	0.487	*S*_23_	84	61

## Data Availability

Not applicable.
